# Anthropometric, physiological characteristics and rugby-specific game skills of schoolboy players of different age categories and playing standards

**DOI:** 10.1186/s13102-019-0155-3

**Published:** 2020-02-10

**Authors:** Matthew Chiwaridzo, Gillian D. Ferguson, Bouwien C. M. Smits-Engelsman

**Affiliations:** 1 Faculty of Health Sciences, Department of Health and Rehabilitation Sciences, Physiotherapy Division, Observatory, University of Cape Town, Cape Town, South Africa; 2 College of Health Sciences, Rehabilitation Department, University of Zimbabwe, P.O Box A178, Avondale, Harare, Zimbabwe

**Keywords:** Adolescents, Rugby union, Anthropometric, Physiological, Rugby-specific game skills

## Abstract

**Background:**

Rugby is increasingly gaining popularity among school-aged male junior players in countries hardly known for dominating international rugby, such as Zimbabwe. Given rugby combativeness, participating adolescents should possess qualities or skills commensurate with the physical demands of the sport for effective participation. This study investigated the independent and interactive effects of age category and playing standard on anthropometric, physiological characteristics and rugby-specific game skills among Zimbabwean athletes.

**Methods:**

Two hundred and eight elite, sub-elite and non-rugby players competing at Under 16 and Under 19 age categories were assessed using the School Clinical Rugby Measure (SCRuM) test battery. Participants underwent height, sitting height, mass, skinfolds, speed, agility, upper-and-lower muscular strength and power, prolonged high-intensity intermittent running ability, tackling, passing and catching assessments in a cross-sectional experimental design.

**Results:**

Age categories had significant main effect on all SCRuM test items except sum of seven skinfolds (*p* = 0.45, η^2^p = 0.003). Playing standard had significant main effects for all variables except height (*p* = 0.40, η^2^p = 0.01) and sum of seven skinfolds (*p* = 0.11, η^2^p = 0.02). Specifically, upper-and-lower muscular strength and power, prolonged high-intensity intermittent running ability, tackling, passing and catching improved with increasing playing standards. However, two-way analysis of variance only demonstrated significant interactions between the effects of age category and playing standards for vertical jump height (VJ) test, 2-kg medicine ball chest throw (2-kg MBCT) test, Yo-Yo intermittent recovery test level 1 (Yo-Yo IRT L1), and tackling and catching tests. Yo-Yo IRT L1, VJ, tackling and catching tests demonstrated greater discriminative ability among Under 16 s, whereas the 2-kg MBCT test showed better ability in Under 19 s.

**Conclusion:**

All SCRuM variables except skinfolds improved with age, highlighting relative sensitivity in differentiating older from younger athletes. However, the discriminative ability by playing standards for VJ, 2-kg MBCT, Yo-Yo IRT L1, tackling and catching ability tests was age-dependent. These findings informs on general attribute development in junior rugby players with age and on specific players attributes in need of monitoring for attainment of elite status at U16 or U19 level.

## Background

Worldwide, burgeoning talent identification (TID) and long-term player development programmes have seen an increased number of male adolescents playing rugby union (rugby, RU) [1, 2]. Regardless of playing standard and age category, adolescent RU is a highly demanding physical and skill-based sport characterised by intermittent execution of high-intensity activities such as sprinting and tackling [3–7]. As such, adolescents playing competitive rugby require well-developed physical or physiological qualities and game skills for effective participation. Accordingly, RU coaches are constantly seeking knowledge on junior players’ attributes linked to elite performance and how these attributes evolve with age for the maintenance of team success.

A plethora of studies have investigated the independent effects of age category or playing standard on test performances of junior rugby players. However, with junior athletes’ performances likely to be determined by the complex interaction of a number of factors such as age and training-related factors, there seems to be limited understanding of the interactive effect of age category and playing standard on development of junior rugby players’ attributes. This knowledge provides insight into the combined effect of age and training efforts on performance differences for rugby players of different playing standards, information which has specific implications on training and player development across various age categories and competitive levels.

Variably, anthropometric, physiological characteristics and game skills have been shown to improve across annual age categories [1, 8–11]. For example, Darrall-Jones et al., [11] showed that body mass and height, but not skinfolds, of elite RU players increased significantly across Under 16 (U16), U18 and U21 age categories. Durandt et al., [8] showed that elite U18 RU players had better scores for upper-body muscular strength and aerobic fitness compared to elite U16 s, but not for speed and agility. Catching and passing-for-accuracy abilities were shown to increase from U16 s to U18 s for elite adolescent RU players [9]. Collectively, most of these studies provide vital information on performance differences of elite RU players across age categories, highlighting the primary influence of age or maturity-related factors in attribute development. The age category differences may allow coaches to monitor development of physical and technical attributes and adopt effective training strategies and programmes that minimise performance gaps between players of different age categories assisting with smooth developmental transition [10]. However, given the possibility that performance differences between younger and older athletes are likely to be related to growth and development-related process regardless of playing standard or sport, the common limitation with these studies has been the lack of a control group possibly including sub-elite rugby players or age-matched male adolescents playing a different sport. Possibly, this would allow for a comparative understanding of the relative effect of age category on performance differences across varying playing standards or sports.

Previous studies investigating the influence of playing standard on RU players attributes have established that anthropometric, physiological characteristics and game skills improve with increasing playing standards [12–16]. Body mass was greater in elite U16 RU athletes from a country known to have higher rugby playing standards compared to elite U16 players derived from a country known to have relatively lesser rugby standards [16]. Jones et al., [12] showed that upper-body muscular strength, 40-m speed, and aerobic fitness contribute to higher playing standard of U18 academy players when compared to lower-level U18 schoolboy rugby players. However, conflicting results have been reported for sum of skinfold thickness measurements [16–20]. In related intermittent sports, lower-body muscular power and agility discriminated U16 elite from sub-elite soccer players [21], whilst elite U16 rugby league (RL) players had better speed, agility, and aerobic capacity compared to sub-elite players [22]. The influence of differing playing standards on player performances may facilitate understanding of specific attributes important for the attainment of elite status, creating a strong foundation for launching targeted training interventions and TID initiatives in junior rugby. Although providing helpful information in identifying important characteristics for elite performance at a distinct age category, the above-cited studies largely assume that junior rugby players’ performances are mainly influenced by differing playing standards or training-related exposures [23], and ignore biological maturation effects, age-related changes and possible interaction effects between age category and playing standards on performance outcomes.

With longitudinal studies hinting on different rates of attribute improvement for players depending on playing abilities and age category [24, 25], it is plausible to hypothesise for significant interactions between age category and playing standard on test performances for athletes. However, it is unclear from previous cross-sectional studies whether age-category differences are similar or different across playing standards and how these differences would compare if competitive rugby players are compared to age-matched non-rugby players playing a different competitive sport. Therefore, comparing anthropometric, physiological characteristics and game skills, the current study examined the independent influence of age category (U19 s vs. U16 s), playing standard (elite vs. sub-elite vs. non-rugby) and the interaction effects (age-category × playing standard) on test performances for young schoolboy athletes. Based mainly on the review findings of Till et al. [19] and specific literature findings on rugby-specific game skills [1, 9], it was hypothesised that:

(i) Anthropometric (except for sum of skinfolds which would remain stable), physiological characteristics and skill ratings would improve with increasing age category.
(ii) Anthropometric, physiological characteristics and rugby-specific game skills would improve with increasing playing standard.
(iii) There would be significant interactions between the effects of age-category and playing standards on test performances.

## Methods

### Study design, research setting and participants

To test study hypotheses, a cross-sectional design was employed to compare participant performances based on the School Clinical Rugby Measure (SCRuM) test battery. The processes involved in developing the test battery have been explained elsewhere [26–30]. Two hundred and eight (208) schoolboys participated in this study and were derived from three different schools. Elite U16 (*n* = 41) and U19 (n = 41) rugby players were recruited from one state school based in Harare, Zimbabwe playing competitive rugby in the SESRL. The SESRL is the most competitive schoolboy rugby league in the country [26]. The school was purposively-selected since they were the defending champions and had won the SESRL thrice in the last five seasons. All sub-elite participants (U16 = 41, U19 = 46) were recruited from a Harare-based private school playing rugby in the CESRL. The CESRL represents a second-tier schoolboy rugby league in Zimbabwe [26]. Cricket players (U16 s = 29, U19 s = 21) represented the non-rugby playing group and were recruited from one of the “top” cricket-playing state high schools based on 2018 provincial inter-scholastic competitions. The justification for including cricket players involved incorporating a second comparative, convenient group of schoolboy athletes playing a competitive sport known to have different physical and technical demands than rugby [31]. All invited players were informed on the study purpose, test procedures, risks and benefits for participating. Ethical approval was sought and granted by the Human Research Ethics Committee (HREC) from the University of Cape Town. Written informed consent and assent were obtained from parents and players, respectively.

### Procedure

All tests were conducted in the order described in Additional file 1, in line with training-related activities. Prior to testing, all eligible participants were familiarised to the test battery items on two consecutive days. Participants either with self-reported injuries precluding physical activity [32] or who partook in multiple sports were excluded. However, injured participants competed in tests they were physically capable of performing. Participants also completed a brief questionnaire soliciting demographic and sport-related information. Data sought included age, sport played, school team, playing experience (number of years since starting training and playing rugby or cricket), number of hours of training per week, regular and alternative positions played, and playing status in the team. All this information had to be corroborated by the head coaches.

A full description of SCRuM test battery is included as Additional file 2. Briefly, the SCRuM had (i) anthropometric (height, sitting height, body mass, seven-site skinfold measurements), (ii) physiological (speed, agility, upper-and-lower muscular strength and power, prolonged high-intensity intermittent running ability, and repeated high-intensity exercise performance ability) and (iii) rugby-specific game skills (tackling, passing, and catching). Only U19 rugby players performed one- repetition maximum bench press (1-RM BP) and back squat (1-RM BS) tests because of regular exposure to resistance training compared to U16 s and cricketers. Instead, 60-s push-up and wall sit leg strength (WSLS) tests were incorporated into the SCRuM for group comparisons on upper-and-lower limb muscular strength, respectively. Inclusion of 60-s push-up test was based on recent findings of a systematic review highlighting common usage of the test for assessment of upper-body muscular strength in junior RU players [27]. The WSLS test is commonly used in training for estimating lower-extremity muscular strength or endurance for adolescent athletes in the local context [26]. Cricket players did not perform repeated high-intensity exercise (RHIE) and rugby-specific game skills due to high-school cricket coaches’ reservations on performing rugby-oriented technical and physical skills.

Since reliability coefficients are population specific [32, 33], elite U16 and U19 rugby players were tested twice in a preliminary study to estimate the absolute and relative reliability of each SCRuM test item. Intraclass correlation coefficients and coefficient of variation for each test item have been presented in previous studies [29, 30]. Baseline data for these players was then compared to data obtained for U16 and U19 sub-elite and non-rugby players. Testing occurred in training during the rugby competitive season (May–August, 2018) for rugby players and cricket competitive season (September–November, 2018) for non-rugby athletes. This timing ensured that participants had gained match-related physical fitness [34, 35]. For each test, participants completed standardised warm-up procedures and were allowed three sub-maximal practice trials following test demonstration by the research assistants. Two trained research assistants conducted all the SCRuM tests, except for skinfolds and game-specific skills which were conducted by subject experts. Testing occurred on natural grass pitch for field tests and the gymnasium was used for strength-and-power based tests. Participants were requested to continue with their normal diet and refrain from caffeine and performance enhancers during the testing period.

### Statistical analyses

The Shapiro Wilk test assessed normality and Levene’s test evaluated equality of error variances for dependent variables (*p* < 0.05). The mean and standard deviation (Mean ± SD) described parametric data. The chi-square test checked for significant differences in proportion for player compositions between elite and sub-elite rugby groups and age categories. Two-way univariate analysis of variance (ANOVA) examined for significant main effect for fixed factors of age category (U16 vs. U19), playing standard (elite vs. sub-elite vs. non-rugby) and whether a significant age category×playing standard interaction existed. In case of significant main effect for playing standards, pairwise comparisons were assessed using Scheffé post-hoc test to locate mean differences. Additionally, identified significant interactions were followed with simple main effect analysis with Bonferroni correction adjusted for multiple comparison tests. Partial eta squared (η^2^p) measured effect size and was interpreted as 0.01 = small, 0.06 = medium and 0.14 = large [36–38]. All analysis were conducted using SPSS version 25.0 with statistical significance accepted when *p* < 0.05.

## Results

Descriptive data on age, playing experience and biological maturation are shown in Table 1. Significant differences between U16 s and U19 s were identified for chronological age, years from peak height velocity (YPHV) and playing experience. There were no significant differences within U16 age category across the playing standard for chronological age and playing experience. However, elite U16 rugby players reached biological maturity significantly earlier compared than sub-elite and non-rugby players. Within U19 age category, no significant differences were observed across playing standards for chronological age, playing experience and YPHV. With regards to player composition, all rugby groups had an equal proportion of forward and back players irrespective of age category [X^2^ (df = 1) =0.00, *p* = 0.99] and playing standards [X^2^ (df = 1) =0.03, *p* = 0.87]. The props and wingers were the majority in both U19 and U16 age categories.

**Table 1:** Sample demographics, biological maturation and sport-related information for each group of participants (*N* = 208)

		U19 Elite	U19 Sub-elite	U19 Non-rugby	All U19 s	U16 Elite	U16 Sub-elite	U16 Non-rugby	All U16 s	*P (df = 5)*
Sample size (*n*)		41	46	21	108	41	30	29	100	
^a^Age (yrs)		17.5 ± 0.85	17.4 ± 0.87	17.6 ± 0.81	17.5 ± 0.85	14.9 ± 0.31	14.8 ± 0.43	14.9 ± 0.28	14.9 ± 0.34	< 0.001†
Age range (yrs)		15.6–18.9	15.7–18.8	15.4–18.9	15.4–18.9	14.4–15.3	13.9–15.3	14.4–15.3	13.9–15.3	
^a^YPHV (years)		1.93 ± 0.53	1.64 ± 0.97	1.78 ± 0.56	1.78 ± 0.76	0.64 ± 0.92	−0.01 ± 0.82	−0.05 ± 0.61	0.24 ± 0.87	< 0.001†§
^a^Playing exp. (years)		4.95 ± 0.74	4.89 ± 0.67	4.74 ± 0.38	4.81 ± 0.74	2.49 ± 0.51	2.23 ± 0.68	2.38 ± 0.56	2.38 ± 0.58	< 0.001†
Generic positions	Forwards, n (%)	21 (51.2)	23 (50.0)	–	44	20 (48.8)	16 (53.3)	–	36	
	Backs, n (%)	20 (48.8)	23 (50.0)	–	43	21 (51.2)	14 (46.7)	–	35	
	Allrounder, n (%)	–	–	10 (47.6)	–	–	–	11 (37.9)	–	
	Batsman, n (%)	–	–	6 (28.6)	–	–	–	11 (37.9)	–	
	Bowler, n (%)	–	–	3 (14.3)	–	–	–	5 (17.2)	–	
	Wicketkeeper, n (%)	–	–	2 (9.52)	–	–	–	2 (6.90)	–	
Specific positions	Props	7	6	–	13	6	5		11	
	Locks	5	7	–	12	3	4	–	7	
	Hookers	3	2	–	5	3	1	–	4	
	Flankers	5	5	–	10	6	4	–	10	
	Eighth man	1	3	–	4	2	2	–	4	
	Scrum half	4	3	–	7	6	3	–	9	
	Fly half	3	3	–	6	2	2	–	4	
	Centres	5	5	–	10	4	3	–	7	
	Wingers	4	9	–	13	7	4	–	11	
	Fullback	4	3	–	7	2	2	–	4	

^a^expressed as M ± SD = mean ± standard deviation; df = degrees of freedom for one way analysis of variance for between group effects; YPHV = years from peak height velocity indicating maturity offset years; n = number; yrs. = years; playing exp. = playing experience representing number of years playing sport in school either rugby or cricket; U=Under; Age-range = minimum year to maximum year

†all U19 groups significantly greater than all U16 groups (*p* < 0.05)

§Elite U16 significantly greater than U16 sub-elite and U16 non-rugby players (*p* < 0.05)

Table 2 depicts mean and standard deviation (M ± SD) scores for anthropometric variables, physiological characteristics and rugby-specific game skills at each age category according to playing standards.

**Table 2:** Anthropometric, physiological characteristics and game skills of elite, sub-elite and non-rugby players by age category

	Under 19 (*n* = 108)			Under 16 (*n* = 100)		
Characteristic	Elite (*n* = 41)	Sub-elite (*n* = 46)	Non-Rugby (*n* = 21)	Elite (n = 41)	Sub-Elite (*n* = 30)	Non-Rugby (*n* = 29)
	Mean ± SD	Mean ± SD	Mean ± SD	Mean ± SD	Mean ± SD	Mean ± SD
Anthropometrics						
Body mass (kg)	77.5 ± 9.58	75.9 ± 11.6	68.5 ± 9.47	63.7 ± 9.09	61.2 ± 15.5	56.1 ± 7.83
Height (m)	1.73 ± 0.06	1.72 ± 0.08	1.71 ± 0.06	1.67 ± 0.08	1.68 ± 0.08	1.66 ± 0.08
Biceps (mm)	6.71 ± 3.62	6.60 ± 3.14	6.57 ± 2.27	5.78 ± 1.70	6.64 ± 1.14	7.00 ± 3.91
Triceps (mm)	9.44 ± 2.95	9.83 ± 4.58	8.36 ± 2.69	9.85 ± 3.25	9.86 ± 1.94	10.8 ± 5.89
Subscapular (mm)	12.8 ± 2.74	13.5 ± 4.64	11.2 ± 2.64	10.9 ± 2.86	11.3 ± 2.70	12.5 ± 6.21
Suprailiac (mm)	8.93 ± 3.84	9.51 ± 3.93	9.52 ± 1.98	8.28 ± 2.97	8.90 ± 2.99	9.97 ± 5.46
Abdomen (mm)	11.4 ± 2.85	13.3 ± 5.90	11.8 ± 2.41	11.4 ± 4.51	12.6 ± 2.86	12.4 ± 6.34
Thigh (mm)	9.98 ± 2.48	11.0 ± 4.83	9.08 ± 2.00	10.7 ± 3.84	11.4 ± 2.29	11.7 ± 4.40
Calf (mm)	5.49 ± 1.03	6.11 ± 2.07	6.17 ± 1.29	6.49 ± 1.55	7.72 ± 1.17	7.73 ± 3.48
Sum of 7 skinfolds (mm)	64.7 ± 15.6	69.8 ± 24.4	62.7 ± 11.6	63.4 ± 17.1	68.4 ± 10.5	72.1 ± 33.1
Physiological tests						
20 m speed (s)	3.25 ± 0.17	3.36 ± 0.23^α^	3.47 ± 0.25	3.50 ± 0.22	3.55 ± 0.22^ƪ^	3.63 ± 0.24
40 m speed (s)	5.60 ± 0.29	5.84 ± 0.40^α^	6.10 ± 0.27	6.14 ± 0.46	6.20 ± 0.60^ƪ^	6.47 ± 0.47
L-run (s)	6.21 ± 0.32	6.33 ± 0.33^α^	6.43 ± 0.25	6.49 ± 0.34	6.62 ± 0.46^ƪ^	6.67 ± 0.27
Vertical jump (cm)	47.8 ± 3.81	42.5 ± 3.84^α^	44.4 ± 3.85	38.3 ± 2.38	34.9 ± 2.82	32.6 ± 4.12
2 kg medicine ball chest throw (m)	9.23 ± 1.26	8.31 ± 1.18	7.18 ± 1.16	6.97 ± 0.64	5.91 ± 0.86	5.83 ± 0.86
60s Push Up (n)	49.7 ± 9.97	43.9 ± 12.0	38.2 ± 6.50	38.4 ± 10.1	35.6 ± 8.90	32.6 ± 7.06
Wall sit leg strength (s)	146.0 ± 9.72	137.5 ± 21.7	132.6 ± 7.41	132.1 ± 6.61	123.3 ± 13.0	121.2 ± 23.0
Yo-Yo IRT (m)	1505.9 ± 75.8	1443.6 ± 259.1^α^	1053.3 ± 148.8	1307.3 ± 228.6	1030.7 ± 269.6	897.9 ± 171.7
1RM back squat (kg)	98.4 ± 14.8	89.5 ± 16.3	–	–	–	–
Relative back squat (kg/kg^−1^)	1.27 ± 0.04	1.17 ± 0.06	–	–	–	–
1RM bench press (kg)	90.5 ± 16.4	80.6 ± 15.9	–	–	–	–
Relative bench press (kg/kg^−1^)	1.16 ± 0.08	1.06 ± 0.06	–	–	–	–
RHIE 1st sprint test (s)	10.2 ± 0.77	10.5 ± 0.81^α^	–	–	–	–
RHIE 2nd sprint test (s)	13.0 ± 1.02	13.2 ± 0.96^α^	–	–	–	–
RHIE 3rd sprint test (s)	16.1 ± 1.49	18.2 ± 1.64^α^	–	–	–	–
RHIE total sprint test (s)	39.3 ± 2.96	41.9 ± 2.97^α^	–	–	–	–
Decrement in RHIE (s)	5.92 ± 1.17	7.76 ± 1.31^α^	–	–	–	–
Rugby-specific tests						
Tackling proficiency (%)	87.9 ± 8.44	84.8 ± 8.16	–	83.0 ± 8.87	68.3 ± 7.94	–
Passing ability (au)	116.2 ± 2.13	113.0 ± 4.07	–	105.9 ± 4.86	104.7 ± 4.34	–
Running-and-catching ability (au)	74.0 ± 1.07	73.5 ± 1.35	–	71.7 ± 2.06	68.3 ± 2.56	–

*Yo-Yo IRT* Yo-Yo intermittent recovery test, *1RM* one repetition maximum, *RHIE* repeated high intensity exercise test measured in seconds, *au* arbitrary units, α sample size was 44, Decrement in RHIE denotes time differences between the first RHIE sprint and last 3rd sprint denoting fatigue time; ƪ = sample size was 26 for the respective tests

Table 3 shows univariate test results for two-way ANOVA. Age category had a significant effect on all dependent variables except sum of seven skinfolds (*p* = 0.45, η^2^p = 0.003). For playing standard, there were significant main effects for all variables except for chronological age (*p* = 0.61, η^2^p = 0.01), height (*p* = 0.40, η^2^p = 0.01) and sum of seven skinfolds (*p* = 0.11, η^2^p = 0.02). Post-hoc analysis revealed that elite and sub-elite rugby groups were significantly better compared to non-rugby players for 20-m speed (*p* < 0.001, η^2^p = 0.09), 40-m speed (p < 0.001, η^2^p = 0.14), 60-s push-up (p < 0.001, η^2^p = 0.11) and WSLS (p < 0.001, η^2^p = 0.13). However, L-run agility scores were significantly better in elite rugby players when compared to non-rugby players (*p* = 0.004, η^2^p = 0.06). Vertical jump (VJ), 2-kg medicine ball chest throw (2-kg MBCT), Yo-Yo IRT L1, tackling proficiency, passing and running-and-catching ability tests improved significantly with increasing playing standards. However, there were significant interactions between age category and playing standard only for: VJ (*p* = 0.01, η^2^p = 0.05), 2-kg MBCT (p = 0.01, η^2^p = 0.04), Yo-Yo IRT L1 (*p* = 0.001, η^2^p = 0.07), tackling proficiency (*p* < 0.001, η^2^p = 0.11) and running-and-catching ability (p < 0.001, η^2^p = 0.14).

**Table 3:** Two-way ANOVA results examining annual age category, playing standards and interaction effects on anthropometrics, physiological and rugby-specific game skills

Characteristic	Age-category				Playing standard				Age-category × playing standard		
	F	P	η^2^p	Comparisons	F	P	η^2^p	Pairwise	F	p	η^2^p
Chronological age (years)	752.2	< 0.001	0.79	U19 s > U16 s	0.50	0.61	0.01	–	0.08	0.92	0.00
Playing experience (years)	642.8	< 0.001	0.76	U19 s > U16 s	4.20	0.02	0.04	E, SE > NR	3.77	0.03	0.04
YPHV (years)	201.2	< 0.001	0.50	U19 s > U16 s	8.08	< 0.001	0.07	E > NR	2.12	0.12	0.02
Anthropometrics											
Body mass (kg)	77.3	< 0.001	0.28	U19 s > U16 s	9.23	< 0.001	0.08	E, SE > NR	0.18	0.84	0.00
Height (m)	26.4	< 0.001	0.12	U19 s > U16 s	0.92	0.40	0.01	–	0.26	0.77	0.00
Sum of skinfolds (mm)	0.56	0.45	0.00	–	2.26	0.11	0.02	–	0.45	0.63	0.00
Physiological tests											
20 m speed test (s)	36.0	< 0.001	0.16	U19 s < U16 s	9.61	< 0.001	0.09	E, SE < NR	0.72	0.49	0.01
40 m speed test (s)	51.1	< 0.001	0.21	U19 s < U16 s	16.1	< 0.001	0.14	E, SE < NR	1.02	0.36	0.01
L-run agility test (s)	31.0	< 0.001	0.14	U19 s < U16 s	5.77	0.004	0.06	E < NR	0.10	0.91	0.00
Vertical jump test (cm)	369.3	< 0.001	0.65	U19 s > U16 s	39.8	< 0.001	0.28	E > SE > NR	5.13	0.01	0.05
2 kg MBCT test (m)	185.4	< 0.001	0.48	U19 s > U16 s	40.2	< 0.001	0.29	E > SE > NR	4.39	0.01	0.04
60s Push Up test (n)	35.7	< 0.001	0.15	U19 s > U16 s	12.4	< 0.001	0.11	E, SE > NR	1.34	0.27	0.01
Wall sit length strength (s)	35.9	< 0.001	0.15	U19 s > U16 s	11.3	< 0.001	0.10	E, SE > NR	0.14	0.87	0.00
Yo-Yo IRT L1 (m)	73.4	< 0.001	0.27	U19 s > U16 s	66.2	< 0.001	0.40	E > SE > NR	7.31	< 0.001	0.07
Game skills											
Tackling proficiency (%) †	62.0	< 0.001	0.29	U19 s > U16 s	43.5	< 0.001	0.22	E > SE	18.3	< 0.001	0.11
Passing ability (au)†	210.4	< 0.001	0.58	U19 s > U16 s	12.5	< 0.001	0.08	E > SE	2.58	0.12	0.02
Running-and-catching ability (au)†	166.9	< 0.001	0.52	U19 s > U16 s	46.7	< 0.001	0.23	E > SE	25.1	< 0.001	0.14

×Interaction; η2p = partial eta squared; E = elite rugby players; SE = sub-elite rugby players; NR = non-rugby players; 2 kg MBCT = 2 kg medicine ball chest throw; Yo-Yo IRT L1 = Yo-Yo intermittent recovery test level 1; YPHV = years from peak height velocity representing maturity offset years; U19 s = Under 19 s; U16 s = Under 16 s; au = arbitrary units; † = 2*2 factorial ANOVA was conducted (age category = U19 vs. U16; Playing standard = elite vs. sub-elite); Pairwise = posthoc test results; One repetition maximum bench press and back squat tests, and repeated high intensity exercise performance ability test are removed from this analysis as there were performed only by U19 rugby athletes and can only be compared between U19 elite and U19 sub-elite

**Fig. 1:**
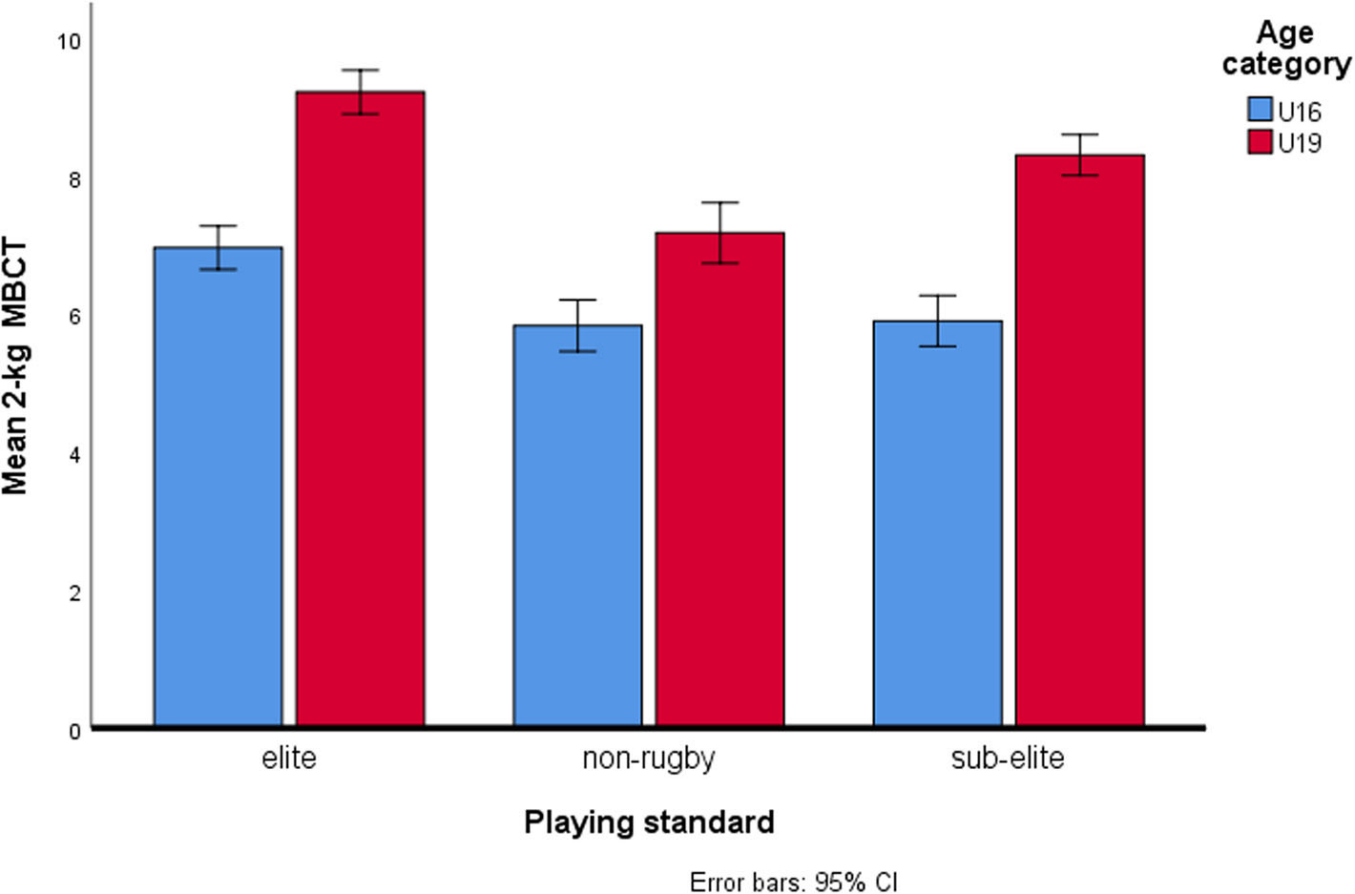
Comparison of 2 kg medicine ball chest throws across playing standards for each age category. There were significant mean differences (*p* < 0.05) in test scores between the U19 s and U16 for elite, sub-elite and non-rugby. For U16 s, 2 kg MBCT test showed good discriminative validity in differentiating elite from both sub-elite and non-rugby players but failed to distinguish sub-elite from non-rugby players. At U19 level, elite rugby players were significantly better than both sub-elite and non-rugby players, and sub-elite were also significantly better from non-rugby players. The largest mean differences between age categories were among the elite and sub-elite

**Fig. 2:**
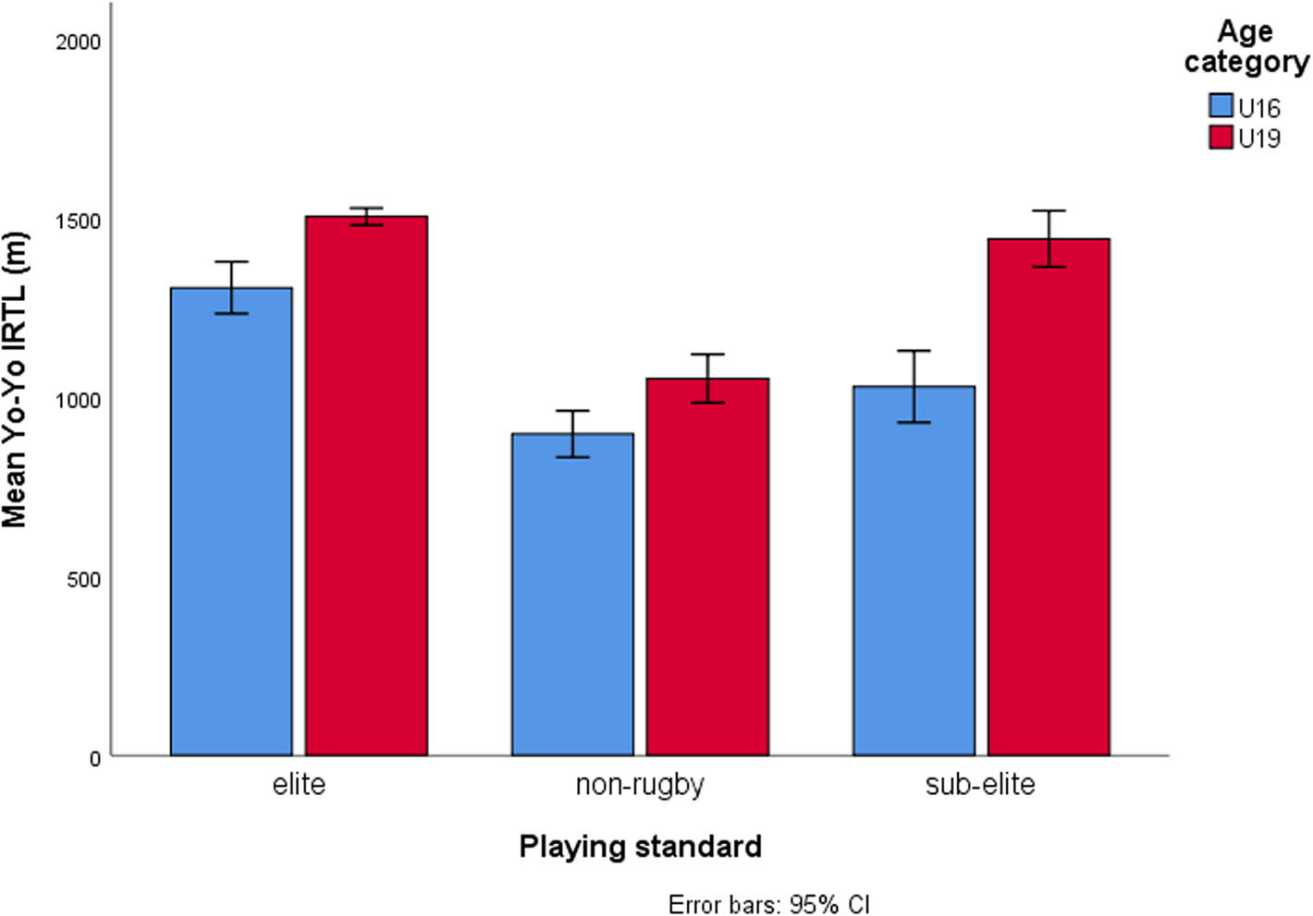
Comparison for Yo-Yo intermittent recovery test across playing standards for the two age-categories. The Yo-Yo IRT L1 test scores significantly improved with increasing playing standard among U16 s but failed to distinguish elite from sub-elite rugby players at U19 level. The sub-elite rugby players showed the largest mean differences between U19 and U16 athletes (p < 0.05; η^2^_p_ = 0.26)

**Fig. 3:**
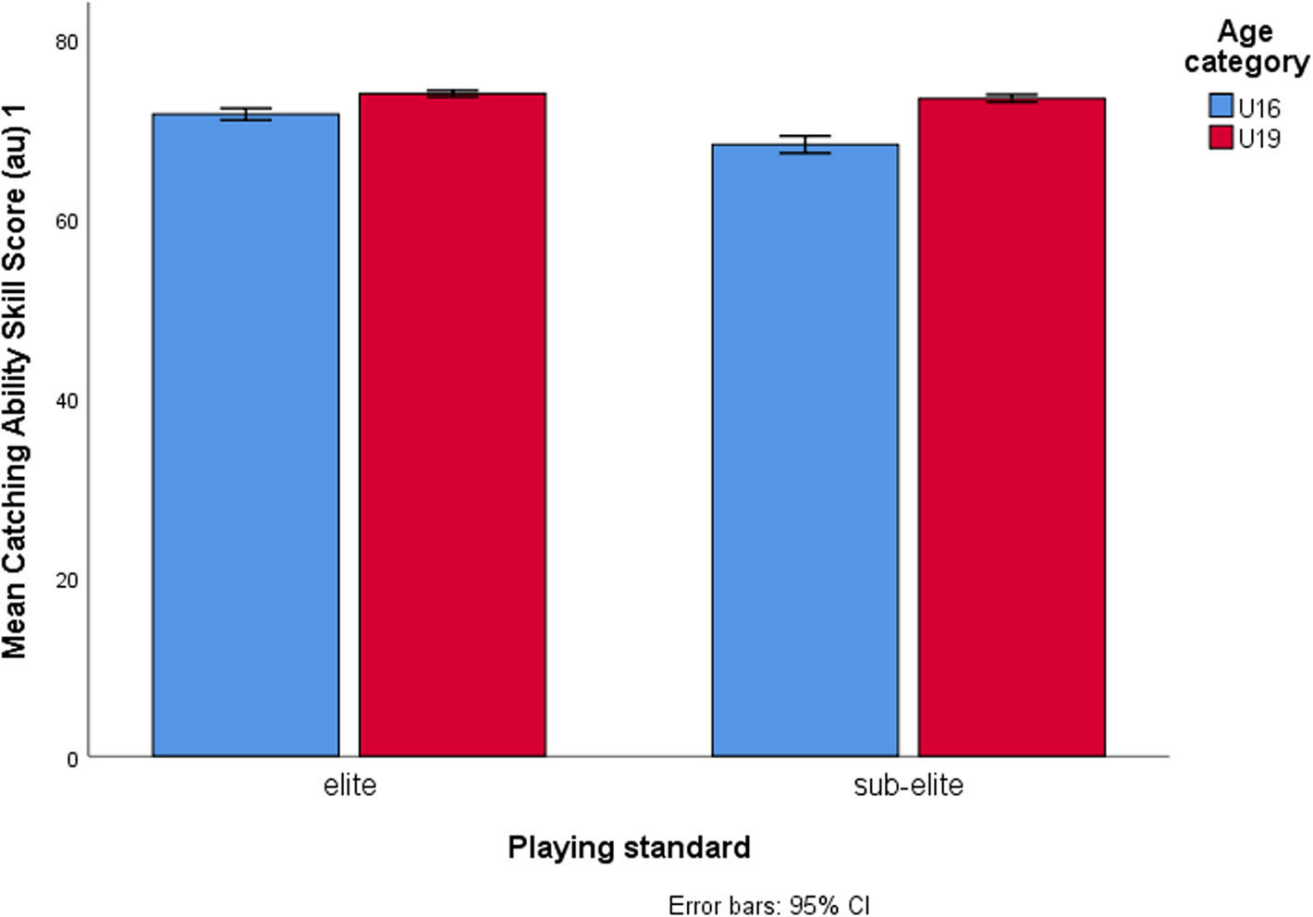
Running-and-catching ability scores compared across playing standards for the U19 and U19 athletes. Elite rugby players outperformed sub-elite rugby players at U16 level and at U19 level there were no significant differences. Greater mean changes between U19 and U16 were among sub-elite rugby players relative to the elite players

**Fig. 4:**
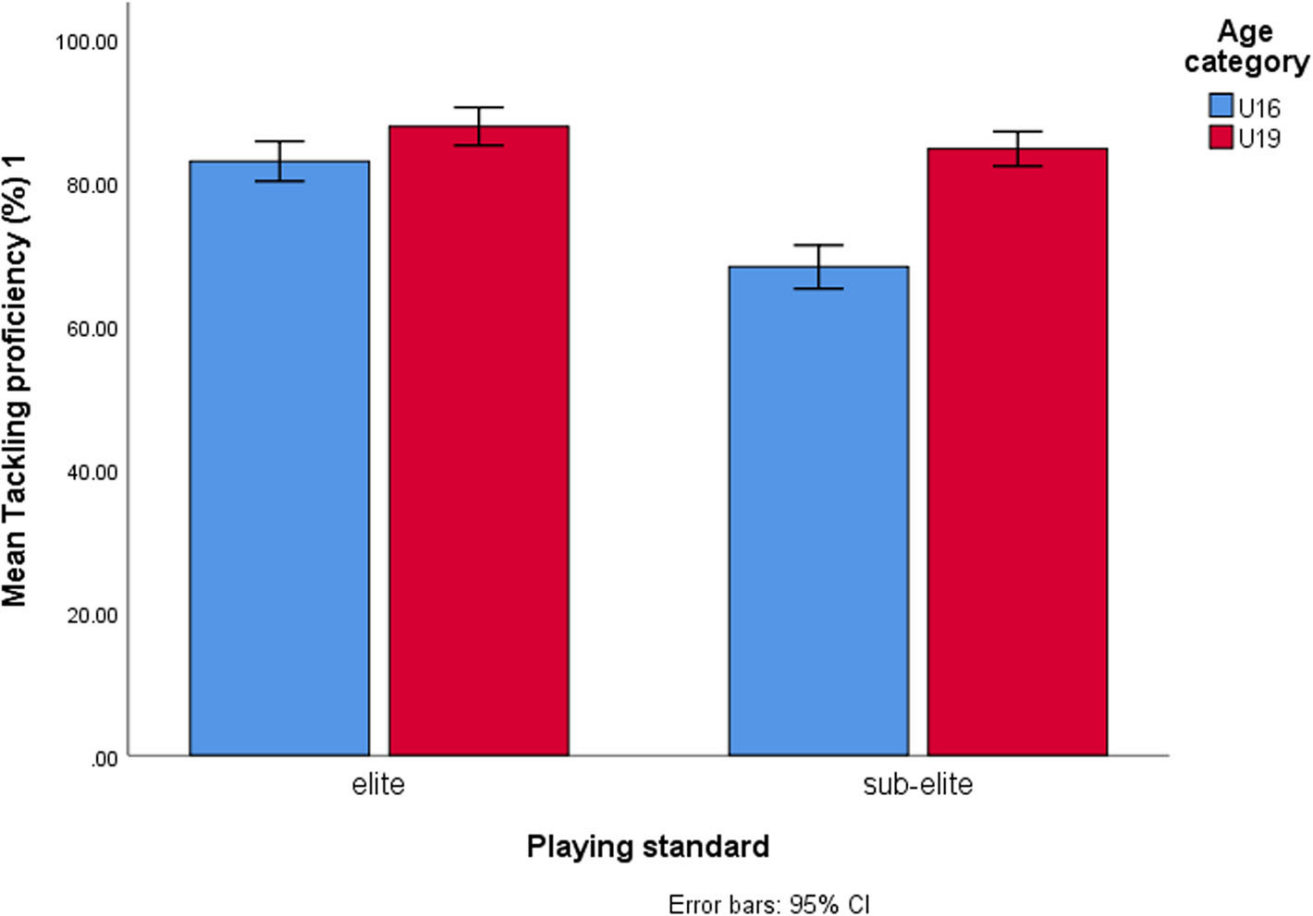
Comparison of elite and sub-elite rugby players for tackling proficiency and age category differences. Elite rugby players significantly outperformed sub-elite rugby players at U16 level and at U19 level there were no significant differences. The sub-elite rugby players showed the largest mean differences between the age categories

**Fig. 5:**
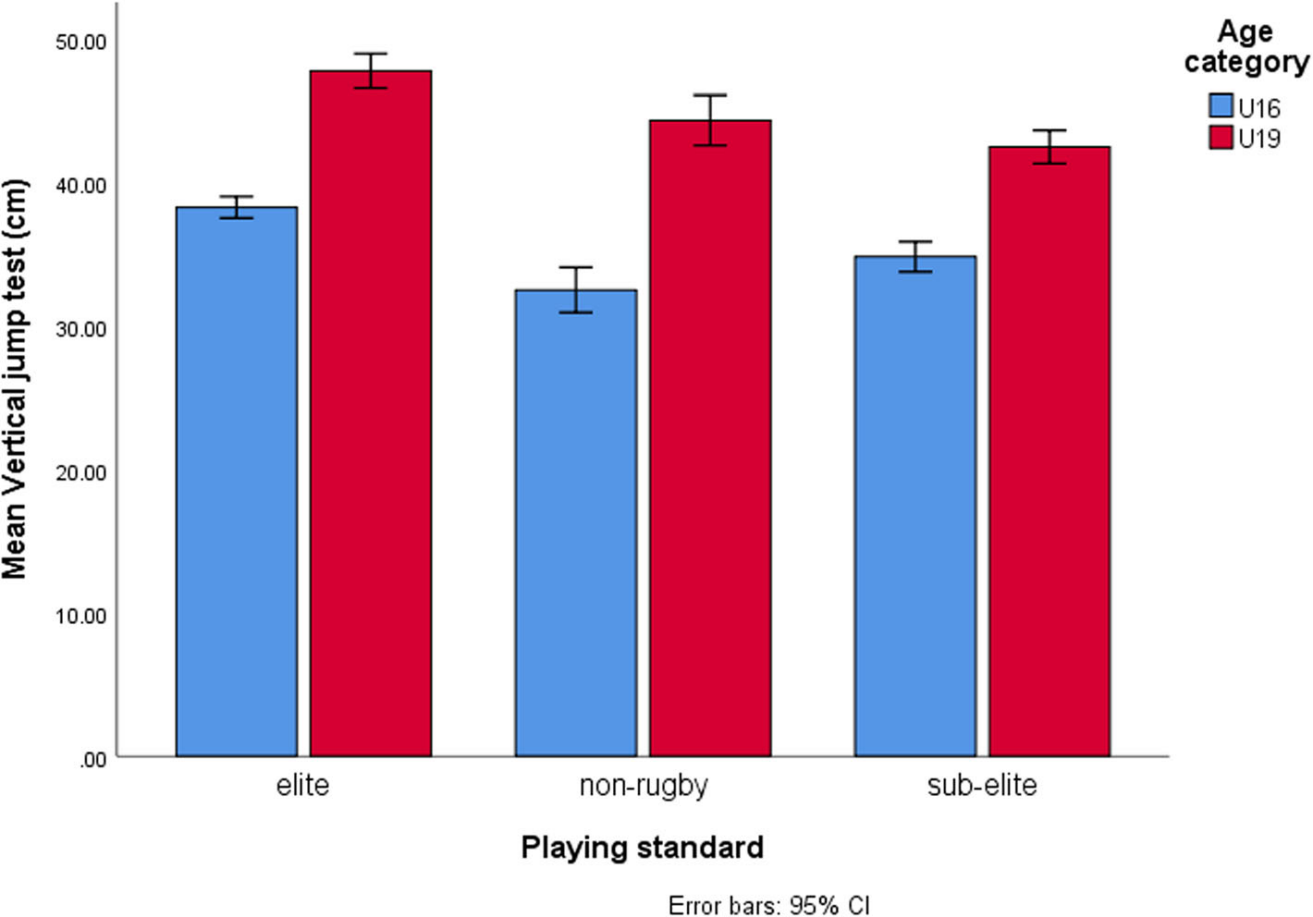
Vertical jump (VJ) test scores. VJ effectively discriminated elite from both sub-elite and non-rugby players and concomitantly sub-elite from non-rugby players at U16 level. At U19 level, non-rugby players showed similar test scores to sub-elite rugby players. The largest mean differences between age categories were among the non-rugby players (p < 0.05; η^2^_p_ = 0.43)

Table 4 displays results for simple main effect analysis indicating mean differences between age-categories across each level of playing standard for dependent variables which showed significant interactions. Between age categories, the largest mean differences in 2-kg MBCT scores (η^2^p = 0.34) (Fig. 1), Yo-Yo IRT L1 (η^2^p = 0.26) (Fig. 2), running-and-catching ability (η^2^p = 0.50) (Fig. 3) and tackling proficiency (η^2^p = 0.31) (Fig. 4) were for sub-elite rugby players. However, non-rugby players showed the largest mean difference for VJ height (η^2^p = 0.43) (Fig. 5).

**Table 4:** Univariate test results for simple main effect analyses of age category on selected dependent variables which showed significant interactions for each level of playing standard

SCRuM test variable	^a^Mean diff (95% CI)	Playing standard	Df	MS	F	P^b^	η^2^p
Running-and-Catching Ability Skill Test (au)	2.27 (1.49–3.05)	Elite group	1	105.476	33.272	< 0.000	0.18
	5.15 (4.32–5.97)	Sub-elite group	1	480.645	151.619	< 0.000	0.50
Tackling Proficiency Test (%)	4.89 (1.22–8.54)	Elite group	1	487.8	6.94	< 0.009	0.04
	16.5 (12.6–20.4)	Sub-elite group	1	4846.4	68.9	< 0.000	0.31
Yo-Yo Intermittent Recovery Test Level 1 (m)	198.5 (168.3–288.7)	Elite group	1	808,043.9	18.8	< 0.000	0.09
	413.0 (316.3–509.7)	Sub-elite group	1	3,042,135.7	70.9	< 0.000	0.26
	155.4 (38.4–272.4)	Non-Rugby group	1	294,145.5	6.86	0.01	0.03
2-kg Medicine Ball Chest Throw Test (m)	2.26 (1.81–2.71)	Elite group	1	104.7	99.6	< 0.000	0.33
	2.41 (1.93–2.88)	Sub-elite group	1	105.1	100.4	< 0.000	0.34
	1.34 (0.77–1.92)	Non-Rugby group	1	22.0	21.0	< 0.000	0.09
Vertical Jump test (cm)	9.52 (7.99–11.0)	Elite group	1	1708.5	140.0	< 0.000	0.41
	7.69 (6.05–9.32)	Sub-elite group	1	1053.9	86.2	< 0.000	0.30
	11.8 (9.87–13.8)	Non-Rugby group	1	1857.7	152.0	< 0.000	0.43

^a^Mean diff = mean differences in the dependent variable between U19 and 16 (Under 19-Under 16) based on estimated marginal means; MS = Mean square; df = degree of freedom; ^b^adjusted for multiple comparisons using Bonferroni correction; η^2^_p_ = partial eta squared; F = each F tests the simple effects of Age category within each level combination of playing standard. These tests are based on the linearly independent pairwise comparisons among the estimated marginal means; 95% CI = 95% confidence interval for the mean difference

## Discussion

This current study showed that age-category had a significant main effect on all SCRuM test items except sum of seven skinfolds. An additional finding was the significant main effect of playing standard without interaction for body mass, 20-m and 40-m speed, L-run, 60-s push-up, WSLS and passing ability skill tests. However, significant interaction effects between age category and playing standard were observed only for VJ, 2-kg MBCT, Yo-Yo IRT L1, tackling proficiency and running-and-catching ability.

As hypothesised and consistent with previous studies [8–10, 20, 27, 34, 39–44], body mass, height, all physiological characteristics and game skills increased with age. These findings provide evidence on the relative sensitivity of these SCRuM test items in effectively discriminating younger rugby and non-rugby participants (U16 s) from older adolescent rugby and non-rugby groups (U19 s). Since U19 s were significantly older compared to U16 s in the present study, age-category differences in anthropometric and test performances could be largely attributed to the normal processes related to growth and maturation that occur during the adolescent period [17, 38, 45, 46]. In the current study, U16 s were, on average, commencing puberty (YPHV = 0.24 ± 0.87 years) whilst U19 s players were approximately 2 years post-peak height velocity (YPHV = 1.78 ± 0.56 years). It is possible that the complex biological events that occur post puberty could explain the observed superior scores for the older participants. Changes in nervous and endocrine systems, muscle and bone morphology, and alterations in metabolism have been reported to be responsible for coordinating anthropometric and physiological alterations [47, 48]. Specifically, large increases in androgens (serum testosterone) concomitantly associated with proliferation of type 2 muscle fibres, muscle hypertrophy (especially in the thighs, calf, upper arms and chest), enhanced neuromuscular firing patterns, and changes in bone length (femur) could collectively explain the higher scores for body mass, stature, muscular upper-and-lower body muscular strength and power, endurance, agility, and speed for U19 s [17, 49]. However, it is also possible that improvement in SCRuM test items with advancing age category could reflect differences in playing experience, training or a combination of the two [8, 42]. For the present study, U19 s had significantly greater playing experience than U16 s and included rugby groups with regular exposure to strength and power resistance training. Resistance training has been shown to increase resting testosterone levels, possibly contributing to the anabolic process during the adolescent growth spurt [47]. In addition, expected higher playing intensities with advancing age, exposure to longer matches (U16 = 60 min vs. U19 = 80 min) and training sessions (U16 = 10 h/week vs. U19 = 15 h/week) for U19 rugby participants may partly explain their superior physiological capacities and better rugby-specific game skills compared to their U16 counterparts.

The present study showed no significant differences for sum of seven skinfolds between U16 s and U19 s age categories. These findings are expected and comparable to related studies [8, 9, 11, 17, 45]. These results were observed despite the significant and large practical differences observed in chronological age, playing experience, biological maturity, body mass and height between U16 s and U19 s. This outcome probably suggests greater stability of skinfolds for schoolboy athletes with increasing age [19] thus dismissing the possible influence of age and the impact of growth processes on skinfold development after U16 age category. In contrast to the study hypothesis that elite rugby players would have a reduced sum of seven skinfolds by virtue of exposure to higher playing intensities, playing standard had no significant main effect on skinfolds. However, in support of these findings, Gabbett et al. [18] also found no significant difference in the sum of seven skinfold thickness between elite and sub-elite players involved in competitive U16 RL. Till et al. [37] also showed no differences among amateur, academy and professional junior RL players albeit at U13 level. A lack of difference in sum of skinfolds has previously been attributed to large interindividual variation within team squads of adolescent groups especially rugby [11], mainly due to the accommodative nature of the sport to all interested school children of various body sizes and shapes.

Although rugby players performed better than non-rugby players, possibly reflecting different speed requirements between rugby and cricket, the present study showed no significant difference in 20-m and 40-m speed tests between elite and sub-elite rugby players. These findings are consistent with previous studies [12] but also contradict others [43]. Speed is regularly listed as an important physiological characteristic in rugby, allowing for players to move fast in attack and defence and has been linked to match success and effective performance of game skills such as tackling [19, 27]. Lack of speed differences between rugby playing standards probably dismisses 20-m and 40-m sprinting abilities as important determinants of higher playing standards in Zimbabwe schoolboy rugby or shows its equal importance in both competitive leagues and the need for continued training. In addition, possible similar exposure to sprinting activities during training [12] and equal proportion of forward and back players in the rugby groups shown in this present study could also account for the lack of difference.

The L-run test failed to discriminate between elite and sub-elite rugby players, and also between sub-elite and non-rugby players. These findings were also shared by previous studies. Gabbett et al. [50] showed that first and second senior grade rugby league players had similar L-run agility scores. Among U16 rugby league players, Gabbett et al. [18] also showed no significant difference in agility scores using the 5–0-5 test between elite and sub-elite rugby league players. The 5–0-5 test utilised in the study by Gabbett et al. [18] involved players performing a speed and agility shuttle run through timing gates. Till et al. [37] also showed similar 5–0-5 agility test scores between academy and professional rugby league players for U13 s, U14 s, and U15 s. Given the reported strong correlation between speed and agility [50], the lack of differences between elite and sub-elite in sprinting shown in the present study could account for the similar agility scores. The significant main effect of playing standard on agility shown in this study emanated from the test validity in differentiating elite players from non-rugby players. Similarly, Till et al. [37] showed that “professional” rugby league players had superior agility test scores compared to the amateurs, however this comparison was for the U14 players. A possible explanation for our finding could be observed differences in speed, playing experience and biological maturity between elite rugby players and non-rugby players.

Greater strength scores were observed for rugby players when compared to non-rugby players. However, there were no significant differences between elite and sub-elite rugby players for the 60-s push-up and WSLS strength tests. There are no studies to the authors’ knowledge that have compared strength performances according to playing standard in junior RU using these tests. However, lack of differences in player composition, maturation, chronological age and playing experience probably explains similar findings for the upper-and-lower muscular strength between elite and sub-elite rugby players. An alternative explanation for the finding could be that these characteristics are equally important for all junior rugby players, irrespective of playing standards. However, when U19 rugby players were assessed for upper-and lower body muscular strength using 1RM BP and 1RM BS, respectively, the results showed a significant difference between the elite and sub-elite players for absolute and relative strength (Table 2). Consistently, Jones et al. [12] showed that professional regional academy U18 RU players representing higher playing standard had superior bench press scores for upper body muscular strength than school-level players. Till et al. [51] also showed that future professional players aged between U17 and U19 had heavier back squat scores when compared with the academy players. However, with the cross-sectional nature of the present study, it is not clear whether our results indicate that stronger U19 schoolboy rugby players are preferentially selected for the elite team resulting in higher measures, or there is increased volume of training muscle strength prevalent in the elite league facilitating greater development of the characteristic when compared to the sub-elite players. It is also possible that both factors could have contributed to this effect. Overall, the present study results expose the poor discriminative validity of both the 60-s push-up and WSLS in differentiating elite and sub-elite rugby players at the U19 level when compared to the 1RM BS and 1RM BP. It suffices, however, to recommend the use of 60-s push-up and WSLS when comparing rugby versus non-rugby players.

Few studies have compared junior rugby players across annual age-categories and playing at different competitive levels for passing ability technical proficiencies. Investigating the relationship between physical fitness and playing ability in rugby league players, Gabbett et al. [23] assessed basic passing based on a skill criteria applied by expert rugby coaches. Similarly, this present study, with a modified passing ability test with eight technical elements for participant evaluation, showed that elite rugby players had superior passing skills compared to sub-elite rugby players. These findings are consistent with previous studies and reflect the importance of passing ability for the attainment of elite status in schoolboy rugby. Gabbett et al. [23] showed that first grade rugby league player had better basic passing skills when compared to third grade players. These differences were attributed to the differences in age (23.7 ± 4.3 years vs. 17.8 ± 1.5 years), and playing experience (16.3 ± 6.7 years vs. 9.4 ± 4.3 years) between the first and third grade players. The present study showed no differences in age, maturity and playing experience between the elite and sub-elite rugby players negating the possible influence of these factors in accounting for the differences observed in the cohort of Zimbabwean schoolboy rugby players. However, with the higher level of proficiency expected in elite rugby and the important role of passing in rugby, it is possible to speculate that enhanced training of pass execution in elite competition is emphasised more than in sub-elite resulting in better passing ability. However, as a limitation, this study did not capture specific details with regards to the actual training content for game skills for rugby players. Future studies may investigate differences in training content by playing standards and see how that influences player performances on game skills such as passing.

The Yo-Yo IRT L1 test scores improved with increasing playing standard among U16 s but failed to distinguish elite from sub-elite rugby players at U19 level. These findings seem to suggest that endurance qualities have a greater impact in determining higher playing standards in U16 RU than in U19 RU. Possibly, increasing playing intensity at U19 level warrants rugby players regardless of playing standard to possess highly developed endurance qualities to cope with the intermittent high-intensity running episodes. However, simple main effect analysis showed greater cross-sectional differences between the age categories for Yo-Yo IRT L1 test scores among sub-elite rugby players. Cognisant of study limitations, these findings possibly indicate heightened endurance training or greater adherence to endurance training activities among U19 sub-elite players compared to U16 sub-elite players resulting in large performance differences between them. On the other hand, relatively small mean difference between U16 s and U19 s was observed for the elite group possibly suggesting robust early onset training of endurance in U16 elite players. Interestingly, young elite U16 s (1307.3 ± 228.6 m) showed similar test performances with sub-elite U19 players (1443.6 ± 259.1 m). These findings suggest that young elite rugby players are reaching older adolescent levels for prolonged high-intensity intermittent running ability relatively faster than either sub-elite or non-rugby players.

At the U16 level, 2-kg MBCT test showed good discriminative validity in differentiating elite from both sub-elite and non-rugby players but failed to distinguish sub-elite from non-rugby players. However, at U19 level, the test effectively discriminated elite rugby players from both sub-elite and non-rugby players, and sub-elite from non-rugby players. With all groups having similar YPHV, age and playing experience, observed differences at U19 level could possibly be accounted for by differences in training strategies across playing standards. Collectively, these findings highlight increasing sensitivity of the 2 kg MBCT test with advancing age in discriminating rugby players by playing standards. Simple main effect analysis showed that larger cross-sectional performance changes in 2-kg MBCT scores between age-categories among rugby players compared to non-rugby players (Table 4). These findings allow for speculation of the importance of upper-body muscular power in rugby relative to cricket, especially among older U19 rugby participants and also hint at the likelihood of greater development with training in rugby regardless of competitive level. Muscular power is essential in rugby for effective tackles and to push opponents when needed [52].

VJ effectively discriminated elite from both sub-elite and non-rugby players and concomitantly sub-elite from non-rugby players at U16 level. However, this changed at U19 level with non-rugby players showing similar test scores to sub-elite rugby players. This happened because there were larger differences in VJ performances with increasing age category for the non-rugby players at U19 level relative to performance differences of other groups. Although the reasons for this are unclear given the cross-sectional design, it is possible to speculate that low physical fitness affect lower body muscular power production among late maturing U16 non-rugby players as evidenced by the low initial test scores relative to other groups. Given similar playing experiences across levels of playing standards at U16 age category, the possibility of specialist training of lower-body muscular power or preferential recruitment of powerful U16 players in the elite and sub-elite rugby groups could explain the relatively higher VJ scores for the rugby players. However, training probably emphasising motor activities such as sprinting and jumping activities that required the production of significant lower-body muscular power could account for the larger performance changes shown by older non-rugby players. These findings may also suggest that elite cricket players may overcome maturational, playing experience and physical fitness disadvantages at U16 level, and develop lower-body muscular power needed for running and jumping for aerial balls to the point of matching sub-elite rugby players with advancing age [37]. Previous longitudinal studies have hinted on relatively weaker athletes having a greater capacity for improvement with advancing age than highly trained athletes [24].

The present study showed a significant interaction between the effects of age-category and playing standard on tackling proficiency and running-and-catching ability. For both tackling and catching, elite rugby players outperformed sub-elite rugby players at U16 level probably suggesting increased sensitivity of these game specific skills in discriminating younger rugby players by playing standards at that level. However, this changed at U19 level with both groups showing no significant differences for both performances, findings which dismiss the usefulness of these skills in differentiating older adolescent rugby players by playing standards. Therefore, between U16 s and U19 s, large differences in the performances of these tests were in sub-elite rugby players compared to the elite rugby players and were shown more for the tackling proficiency test. The reasons for these findings are unclear given the observational nature of the present study and require further testing in future studies. The low initial performances of sub-elite U16 rugby players relative to elite U16 rugby players possibly reflecting poor training or less proficiency in skill execution especially for tackling could account for the large performance gaps between U16 s and U19 s for the sub-elite group. Alternatively, greater adaptation to training of tackling and catching with increasing age, maturity, playing experience and playing intensity among sub-elite players could also explain the seemingly better performances at U19 level. For tackling, it seems that elite U16 rugby players reach top level scores early as evidenced by relatively small mean differences with the elite U19 rugby group. These findings probably indicate that young elite U16 rugby players reach mature level scores for tackling early than sub-elite rugby players suggesting either greater proficiency or less adaption to training in elite players than in sub-elite rugby players.

### Critical assessment of the study

Novelty in the current study was highlighted by comparing elite, sub-elite and non-rugby players at U16 and U19 age-categories from a country hardly known for dominating international rugby events. However, this study has limitations and the results should be interpreted cautiously in light of these limitations.

- The study involved purposive selection of single schools to represent each playing standard and included only U16 s and U19 s to represent young and older adolescent athletes. This sample may not have been representative of all age-categories and the multiple schools competing in the SESRL, CESRL and cricket interscholastic competitions in the country. The anthropometric, physiological and game skills are likely to differ with chronological age, schools, training strategies, player selection criteria, and player motivation and coaching philosophies possibly over-or under-estimating the fitness, body composition or skills of junior elite and sub-elite players [53]. This limits the external validity of study results to other schools not involved in the study and also to other age-categories not assessed in this study.
- Given the complexity and multifaceted nature of the sport of rugby, only examining the anthropometric, physiological and game specific skills is a possible limitation and a more holistic protocol including tactical, perceptual-cognitive skills and psychological measures would have been ideal to comprehensively understand and identify qualities or skills discriminating players of different ages and playing standards [37]. A recent study showed that psychological attributes such as players’ attitudes and personality traits, mental strength and emotional stability are key qualities that coaches consider in good adolescent rugby players and in player recruitment for TID initiatives [54]. Further studies objectively assessing these qualities and how they differ with age and playing standards in junior rugby are warranted.
- The cross-sectional nature of the study lacked analysis over an extended period of time [38]. This design ignores the dynamic nature of player development possibly narrowing the usefulness of the data for TID [55]. However, the data are crucial for hypothesis generation which could be further tested in future prospective cohort studies. Also, the sample size was limited to allow for the categorisation of participants by player positions.

## Conclusion

This is the first Zimbabwean study to compare anthropometric, physiological characteristics and rugby-specific game skills of schoolboy rugby players (including non-rugby players as a comparative group) of different age categories and playing standards. All anthropometric, physiological characteristics and game skills progressively increased with age except for sum of seven skinfolds suggesting large influence of age and maturity-related factors on attribute development among schoolboy athletes. With regards to playing standards, upper-and-lower-body muscular power, prolonged high intensity intermittent running ability, tackling, passing, running-and-catching ability improved with increasing playing standards. However, there were significant interactions between the effects of age category and playing standard for upper-and-lower-body muscular power, prolonged high intensity intermittent running ability, tackling and catching. These findings suggest that, for these variables, the discriminative ability for playing standard is dependent on age category. Yo-Yo IRT L1, VJ, tackling and catching tests demonstrated greater discriminative ability among Under 16 s than in Under 19 s whilst the 2-kg MBCT test showed the converse. From a practical perspective, Yo-Yo IRT L1, VJ, tackling and catching tests could be used as screening tests for talent search in young rugby players whilst the 2-kg MBCT test is sensitive in differentiating older male adolescent players by playing standards.

## Supplementary information

**Additional file 1.** Order of the SCRuM tests performed during reliability study and subsequent testing of the participants.

**Additional file 2.** The SCRuM test battery.

## Data Availability

The datasets generated and/or analysed during the current study are not publicly available due to the fact that the data is part of ongoing research. However, the data are available from the corresponding author on reasonable request.

## Abbreviations

1RM BP: One repetition maximum bench press
1RM BS: One repetition maximum back squat
2-kg MBCT: 2-kg Medicine Ball Chest Throw
ANOVA: analysis of variance
CESRL: Co-educational School Rugby League
HREC: Human Research Ethics Committee
RL: Rugby League
RU: Rugby Union
SCRuM: School Clinical Rugby Measure
SESRL: Super Eight School Rugby League
TID: Talent identification
U: Under
VJ: Vertical Jump
WSLS: Wall Sit Leg Strength
Yo-Yo IRT L1: Yo-yo Intermittent Recovery Test Level 1
YPHV: Years from peak height velocity
