# Supplementary material for: Anthropometric, physiological characteristics and rugby-specific game skills of schoolboy players of different age categories and playing standards
Source: BMC Sports Sci Med Rehabil. 2020 Feb 10;12:3. doi: 10.1186/s13102-019-0155-3 (PMC7008540; doi:10.1186/s13102-019-0155-3)
Supplement: Supplementary file 1 — Additional file 1. Order of the SCRuM tests performed during reliability study and subsequent testing of the participants. [file 13102_2019_155_MOESM1_ESM.docx]

**Additional file 1: Order of the SCRuM tests performed during test-retest reliability study and subsequent testing of the participants**

| **Group** | ***Week** | **Mon** | **Tues** | **Wedn** | **Thurs** | **Fri** | **Sat** | **Sun** |
| --- | --- | --- | --- | --- | --- | --- | --- | --- |
| E U19 | Week 1 | Body mass | Yo-Yo | 1RM BP | Speed | RHIE | Match | Rest |
|  |  | Height | 2kg MBCT | WSLG | SR | Push Up |  |  |
|  |  | 7 Skin folds |  | 1RM BS | L-run |  |  |  |
|  |  | Sitting height |  | VJ |  |  |  |  |
|  |  |  |  |  |  |  |  |  |
|  | Week 2 | Body mass | Yo-Yo | 1RM BP | Speed | RHIE | Match | Rest |
|  |  | Height | 2kg MBCT | WSLG | SR | Push Up |  |  |
|  |  | 7 Skin folds |  | 1RM BS | L-run |  |  |  |
|  |  | Sitting height |  | VJ |  |  |  |  |
|  |  |  |  |  |  |  |  |  |
|  | Week 3 | Tackling | Passing |  | Catching |  | Match | Rest |
|  |  |  |  |  |  |  |  |  |
|  | Week 4 | Tackling | Passing |  | Catching |  | Match | Rest |
| ***Familiarisation of SCRuM test items to Sub-Elite U19 rugby players*** | | | | | | | | |
| SE U19 | Week 6 | Body mass | Yo-Yo | VJ | Speed | RHIE | Match | Rest |
|  |  | Height | 2kg MBCT | WSLG | SR | Push Up |  |  |
|  |  | 7 Skin folds |  | 1RM BS | L-run |  |  |  |
|  |  | Sitting height |  | 1RM BP |  |  |  |  |
|  |  |  |  |  |  |  |  |  |
|  | Week 7 | Tackling | Passing |  | Catching |  | Match | Rest |
|  | ***Week 8-9*** | ***Familiarisation of SCRuM test items to Elite U16 rugby players*** | | | | | | |
| E U16 | Week 10 | Body mass | Yo-Yo | VJ | Speed | Push Up | Match | Rest |
|  |  | Height | 2kg MBCT | WSLG | SR |  |  |  |
|  |  | 7 Skin folds |  |  | L-run |  |  |  |
|  |  | Sitting height |  |  |  |  |  |  |
|  |  |  |  |  |  |  |  |  |
|  | Week 11 | Body mass | Yo-Yo | VJ | Speed | Push Up | Match | Rest |
|  |  | Height | 2kg MBCT | WSLG | SR |  |  |  |
|  |  | 7 Skin folds |  |  | L-run |  |  |  |
|  |  | Sitting height |  |  |  |  |  |  |
|  |  |  |  |  |  |  |  |  |
|  | Week 12 | Tackling | Passing |  | Catching |  | Match | Rest |
|  |  |  |  |  |  |  |  |  |
|  | Week 13 | Tackling | Passing |  | Catching |  | Match | Rest |
|  | ***Week 14-15*** | ***Familiarisation of SCRuM test items to sub-elite U16 rugby players*** | | | | | | |
| SE U16 | Week 16 | Body mass | Yo-Yo | VJ | Speed | Push Up | Match | Rest |
|  |  | Height | 2kg MBCT | WSLG | SR |  |  |  |
|  |  | 7 Skin folds |  |  | L-run |  |  |  |
|  |  | Sitting height |  |  |  |  |  |  |
|  |  |  |  |  |  |  |  |  |
|  | Week 17 | Tackling | Passing |  | Catching |  | Match | Rest |
|  | ***Week 1-2*** | ***Familiarisation of SCRuM test items to U19 cricket players*** | | | | | | |
| U19 Cr | Week 3 | Body mass | Yo-Yo | WSLG | Speed | Push Up | Match | Rest |
|  |  | Height | 2kg MBCT | VJ | SR | L-run |  |  |
|  |  | 7 Skin folds |  |  |  |  |  |  |
|  |  | Sitting height |  |  |  |  |  |  |
|  |  |  |  |  |  |  |  |  |
|  | Week 4 |  | Passing |  | Catching |  | Match | Rest |
|  | ***Week 5-6*** | ***Familiarisation of SCRuM test items to U16 cricket players*** | | | | | | |
| U16 Cr | Week 7 | Body mass | Yo-Yo | WSLG | Speed | Push Up | Match | Rest |
|  |  | Height | 2kg MBCT | VJ | SR | L-run |  |  |
|  |  | 7 Skin folds |  |  |  |  |  |  |
|  |  | Sitting height |  |  |  |  |  |  |
|  | Week 8 |  | Passing |  | Catching |  | Match | Rest |

_* represents the time the testing commenced which was exactly 3 weeks after the inception of the SESRL. Yo-Yo=Yo-Yo Intermittent Recovery Level 1 Test; 2kg MBCT=2kg medicine ball chest throw tests; 1RM BP=One repetition maximum bench press test; 1RM BS=One repetition maximum back squat test; WSLG=Wall Sit Leg Strength test; VJ=Vertical Jump test; SR-Sit-and-Reach test; Push Up=60s push up test; RHIE=Repeated High Intensity Exercise Performance Ability test; Match=Represents competitive match; 7 skin folds=biceps, triceps, subscapular, suprailiac, abdomen, thigh, and calf measures. Tackling=Tackling proficiency test; Passing=Passing ability and passing for accuracy for 7m test; Catching=Running and Catching Ability test. E=Elite, SE=sub-elite, Cr=Cricket; U=under;_
